# Quantifying the spatial clustering characteristics of radiographic emphysema explains variability in pulmonary function

**DOI:** 10.1038/s41598-023-40950-8

**Published:** 2023-08-24

**Authors:** Brian E. Vestal, Debashis Ghosh, Raúl San José Estépar, Katerina Kechris, Tasha Fingerlin, Nichole E. Carlson

**Affiliations:** 1 Center for Genes, Environment and Health, National Jewish Health, Denver, CO USA; 2 Department of Biostatistics and Informatics, University of Colorado Denver, Anschutz Medical Campus, Aurora, CO USA; 3 Applied Chest Imaging Laboratory (ACIL), Brigham and Women’s Hospital, Harvard Medical School, Boston, MA USA

**Keywords:** Image processing, Statistical methods, Diagnostic markers, Chronic obstructive pulmonary disease

## Abstract

Quantitative assessment of emphysema in CT scans has mostly focused on calculating the percentage of lung tissue that is deemed abnormal based on a density thresholding strategy. However, this overall measure of disease burden discards virtually all the spatial information encoded in the scan that is implicitly utilized in a visual assessment. This simplification is likely grouping heterogenous disease patterns and is potentially obscuring clinical phenotypes and variable disease outcomes. To overcome this, several methods that attempt to quantify heterogeneity in emphysema distribution have been proposed. Here, we compare three of those: one based on estimating a power law for the size distribution of contiguous emphysema clusters, a second that looks at the number of emphysema-to-emphysema voxel adjacencies, and a third that applies a parametric spatial point process model to the emphysema voxel locations. This was done using data from 587 individuals from Phase 1 of COPDGene that had an inspiratory CT scan and plasma protein abundance measurements. The associations between these imaging metrics and visual assessment with clinical measures (FEV$_1$, FEV$_1$-FVC ratio, etc.) and plasma protein biomarker levels were evaluated using a variety of regression models. Our results showed that a selection of spatial measures had the ability to discern heterogeneous patterns among CTs that had similar emphysema burdens. The most informative quantitative measure, average cluster size from the point process model, showed much stronger associations with nearly every clinical outcome examined than existing CT-derived emphysema metrics and visual assessment. Moreover, approximately 75% more plasma biomarkers were found to be associated with an emphysema heterogeneity phenotype when accounting for spatial clustering measures than when they were excluded.

## Introduction

Chronic Obstructive Pulmonary Disease (COPD) is a progressive disease of the lungs that is estimated to affect over 500 million people globally and is the third leading cause of death in the United States^1–3^. Two complementary disease processes drive COPD: small airway disease and pulmonary emphysema. In this work we focus on emphysema where radiographic diagnosis typically relies on visual assessment of chest Computed Tomography (CT) scans, but this requires access to trained assessors, is generally time consuming, and can have poor inter-rater reliability for the consistency of reads^4–7^. Because of these limitations, there has been substantial interest in developing quantitative measures directly from the CT scan. Indeed, the conduct of clinical trials for novel treatments would greatly benefit from augmenting visual assessment with additional objective and reproducible biomarkers of disease subpopulations in order to better classify subjects more likely to share molecular mechanisms of disease, and thus demonstrate a greater or lesser likelihood to respond to a particular treatment^8–10^.

Most research into quantitative measures of emphysema have focused on computing a percentage of the lungs that is determined to be emphysematous^5,6,11–13^. Identification of diseased tissue has generally been done by comparing the observed radiodensity of the lung tissue, as measured in Hounsfield Units (HU), in an inspiratory scan to a threshold (typically -950 HU), and then all voxels with an observed HU less than that threshold are determined to be Low Attenuation Areas (LAAs)^6^. The percentage of all lung voxels that are LAAs (%LAA) is used as the quantitative summary for each subject’s lungs. This can be done at a global scale, or at a regional level (e.g., in the individual lobes), and %LAA has been shown to associate with relevant measures like Forced Expiratory Volume in 1 second (FEV$_1$) and Forced Vital Capacity (FVC)^6,14,15^. However, using %LAA likely collapses heterogeneous disease subtypes because it is a simple measure of severity that discards virtually all the spatial information available in the CT scan that implicitly goes into a visual assessment. Indeed, emphysema itself is a heterogeneous disease process with several subtypes (i.e. centrilobular, panlobular, and paraseptal) that are in-part defined by different spatial characteristics^16,17^. This limitation of %LAA to capture relevant information about distribution and pattern of disease has been previously noted by, for example, Kirby et al.^18^ who found that %LAA and visual assessment contained complementary information when explaining pulmonary function.

To address this problem, several different methods for quantifying spatial heterogeneity of emphysema distribution have been proposed. One of the early methods described in Mishima et al.^19^ investigated the size distribution of LAA clusters in CT scans using a fractal geometry approach . The authors demonstrated that the size distribution of contiguous LAA clusters (LACs) in 2D axial slices followed a power law distribution where the exponent D is used as a corollary to the fractal dimension of the terminal airspaces in that slice. Numerous subsequent follow-ups have demonstrated associations with, among other things, pulmonary function, disease progression, and mortality^20–23^. However, this method has several notable limitations that include relying on connected components analysis to define clusters, including single LAA voxels as clusters, and the emergence of “super clusters” in the 3D version of this analysis in scans with increasing %LAA that potentially break the power law relationship^21^. Another method proposed by Virdee et al.^24^ uses join-count statistics to quantify the compactness of LAA voxels. This is done by counting the number of LAA-to-LAA voxel adjacencies in a given CT scan, and they showed this value, termed the Normalized Join-Count (NJC), is associated with various measures of pulmonary function independent of %LAA and Mishima’s D. This method also relies on a similar connected components framework since only the immediate neighbors or each voxel are considered when counting joins, and thus it may suffer from some of the same limitations as the power law exponent method.

The final method we focus on is a spatial point process framework for analyzing LAAs in chest CT scans originally developed in Vestal et al.^17^. This entailed fitting a hierarchical shot-noise Cox Process to the locations of LAA voxels and then estimating several clustering characteristics of the LAAs. In the original paper, the authors focused on the formal development of the point process model and parameter estimation techniques, and only demonstrated differences in selected clustering measures between scans from various visual assessment subtypes in a smaller set of patients. We further expand upon that work by establishing variability in clustering characteristics between individuals with similar %LAA values, and then showing how they relate to relevant pulmonary function measures.

In the remainder of this paper, our goal is to illustrate how these various emphysema quantification methods compare to each other in their ability explain variation in clinically-relevant patient outcomes, and then use those results to recommend how one can generate the strongest emphysema phenotypes by using some combination of these measures. Utilizing a well-characterized dataset of approximately 600 subjects from the COPDGene study^25^, we examined relationships between these imaging metrics for quantifying spatial heterogeneity of emphysema distribution, visual assessment of emphysema, clinical outcomes, and plasma protein abundance levels using standard regression modeling approaches.

## Methods

### Study population

All data used in this study comes from participants enrolled in Phase 1 of COPDGene, which is a prospective multicenter observational study designed to identify genetic factors associated with COPD^25^. Between 2008-2011, 10,192 cigarette smokers were enrolled in the first phase of this HIPPA-compliant study at 20 centers across the United States where institutional review board approval was obtained at each of: Ann Arbor VA Medical Center, Baylor College of Medicine, Brigham and Women’s Hospital, Columbia University Medical Center, Duke University Medical Center, Johns Hopkins University, L.A. Biomedical Research Institute, Minneapolis VA Medical Center, Minnesota Health Partners - Twin Cities, Morehouse School of Medicine, National Jewish Health, Reliant Medical Group (Fallon), Temple University, University of Alabama, Birmingham, University of California, San Diego, University of Iowa, University of Michigan, University of Minnesota, University of Pittsburgh, and University of Texas, Health San Antonio. Written informed consent was obtained from each participant, and the image analysis methods described here were all carried out with in accordance to relevant guidelines and regulations. Collection of clinical and imaging characteristics for these individuals have been previously described^25,26^. We utilized a subset of 587 individuals that were chosen because they had an inspiratory CT scan that passed quality control, spirometery data, and a plasma protein array as detailed in Carolan et al.^27^; a summary of this population is presented in Table 1.

**Table 1:** Summary of the COPDGene patient subset used in this study.

	Mean or count	SD or %
Sex		
Male	301	51%
Female	286	49%
Cur.Smoker		
No	440	75%
Yes	147	25%
Age		
	63.5	8.6
BMI		
	28.3	5.6
FEV$_1$		
	2.02	1.03
FVC		
	3.34	1.02
FEV$_1$-FVC Ratio		
	0.59	0.20
6-min Walk Dist.		
	1461	375
SGRQ Score (Total)		
	27.9	23.1
FRC		
	3.58	1.22
FRC-TLC Ratio		
	0.57	0.11
GOLD Stage		
PRISm	10	2%
0	236	40%
1	7	1%
2	140	24%
3	123	21%
4	71	12%

*FEV*$_1$ forced expiratory volume in one second, *FVC* forced vital capacity, *6MWD* 6-minute walk distance, *FRC* functional residual capacity, *TLC* total lung capacity, *GT* gas trapping.

### Quantitative image analysis

**Figure 1:**
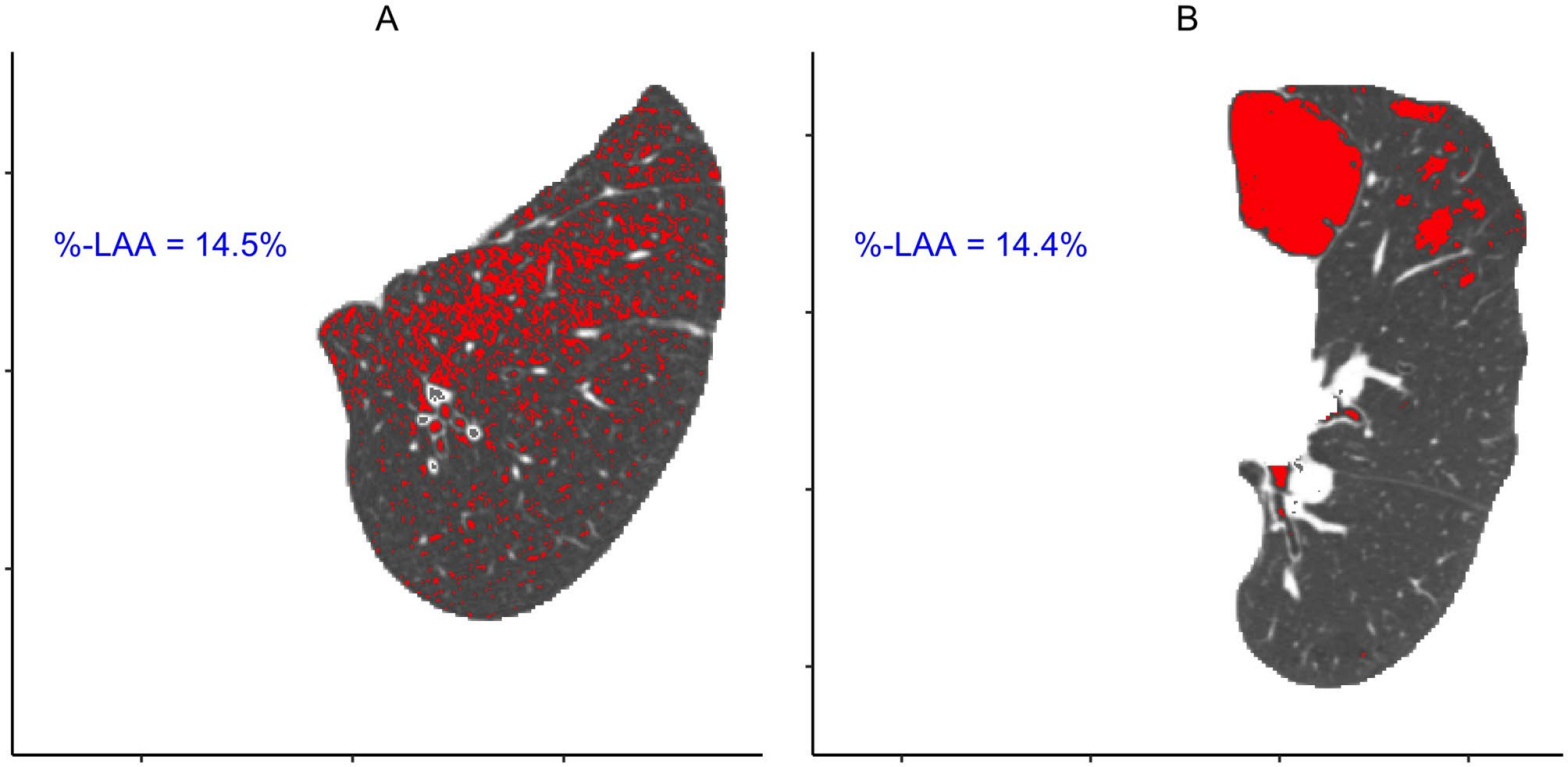
Two axial CT slices of lung tissue with nearly identical %LAA, but very different spatial distributions of disease. *LAA* low attenuation area.

In COPDGene, volumetric inspiratory and expiratory scans were obtained at each visit using a standardized protocol^14,25^. All scans were acquired at 120 kVp, and the scans were reconstructed with a slice thickness of 0.625 mm or 0.75 mm depending on the manufacturer of the scanner. To achieve nearly isotropic voxels, slice intervals were 0.625 mm and 0.50 mm for the two respective voxel heights. Of the 587 CT scans used for this study, 562 (96%) had the latter combination of voxel height and interval, while just 25 (4%) had the former. As part of the COPDGene study, lung and airway segmentations were generated using the Thirona lung quantification software (Thirona, the Netherlands, http://www.thirona.eu) and visually approved by trained analysts. Within the segmented lungs, all of the emphysema quantification methods (Table 2) rely on first generating a binary mask which identifies which voxels are LAAs. To do this, we used the thresholding technique described above where any voxel with a HU$<-950$ was considered an LAA. The most basic measure of quantitative emphysema, %LAA, was computed for each scan by dividing the number of LAA voxels by the total number of lung voxels. Figure 1 shows two axial CT slices with the binary LAA masks overlaid on the HU values. Note that these two slices have virtually identical %LAA, but very different spatial distributions of diseased tissue.

**Table 2:** Summary of the quantitative emphysema measures and their physical units.

CT measure	Units	Mean	SD
%LAA	%	9.52	11.66
Mishima’s D	Unitless	1.70	0.12
NJC	%	7.10	10.05
ACS	mm$^2$	21.35	19.75
NC	Rate per 100 cm$^2$ of lung tissue	17.10	12.76
%-Diffuse	%	29.35	20.13
ACA	mm$^2$	60.80	29.30

*LAA* low attenuation area, *NJC* normalized join-count, *ACS* average cluster size, *NC* number of clusters, *ACA* average cluster area.

#### Power law exponent D

**Figure 2:**
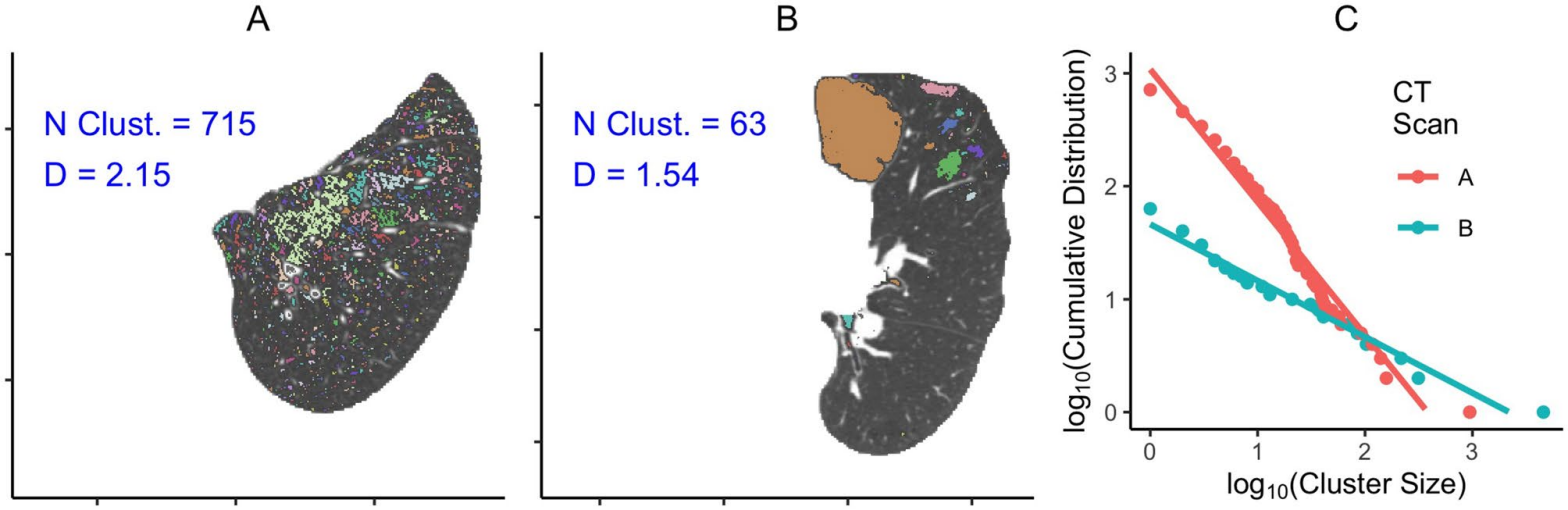
Panels (**A**) and (**B**) show the connected components clustering of the LAAs. Panel (**C**) shows relationship between cluster size and the cumulative distribution function for these two slices, and then the power law exponent D is approximately equal to the negative of the slope from the fitted lines.

Using the 3D locations of LAA voxels for an individual scan, contiguous LACs were identified using the *connected.pp3()* function within the spatstat R package and the individual cluster sizes were recorded. A power law model was then fit using the *fit_power_law()* function from the igraph R package using the maximum likelihood approach to obtain the value of D for that scan. The connected components clustering and estimated power law exponents for the two example 2D slices from Fig. 1 are shown in Fig. 2.

#### Normalized join-count

**Figure 3:**
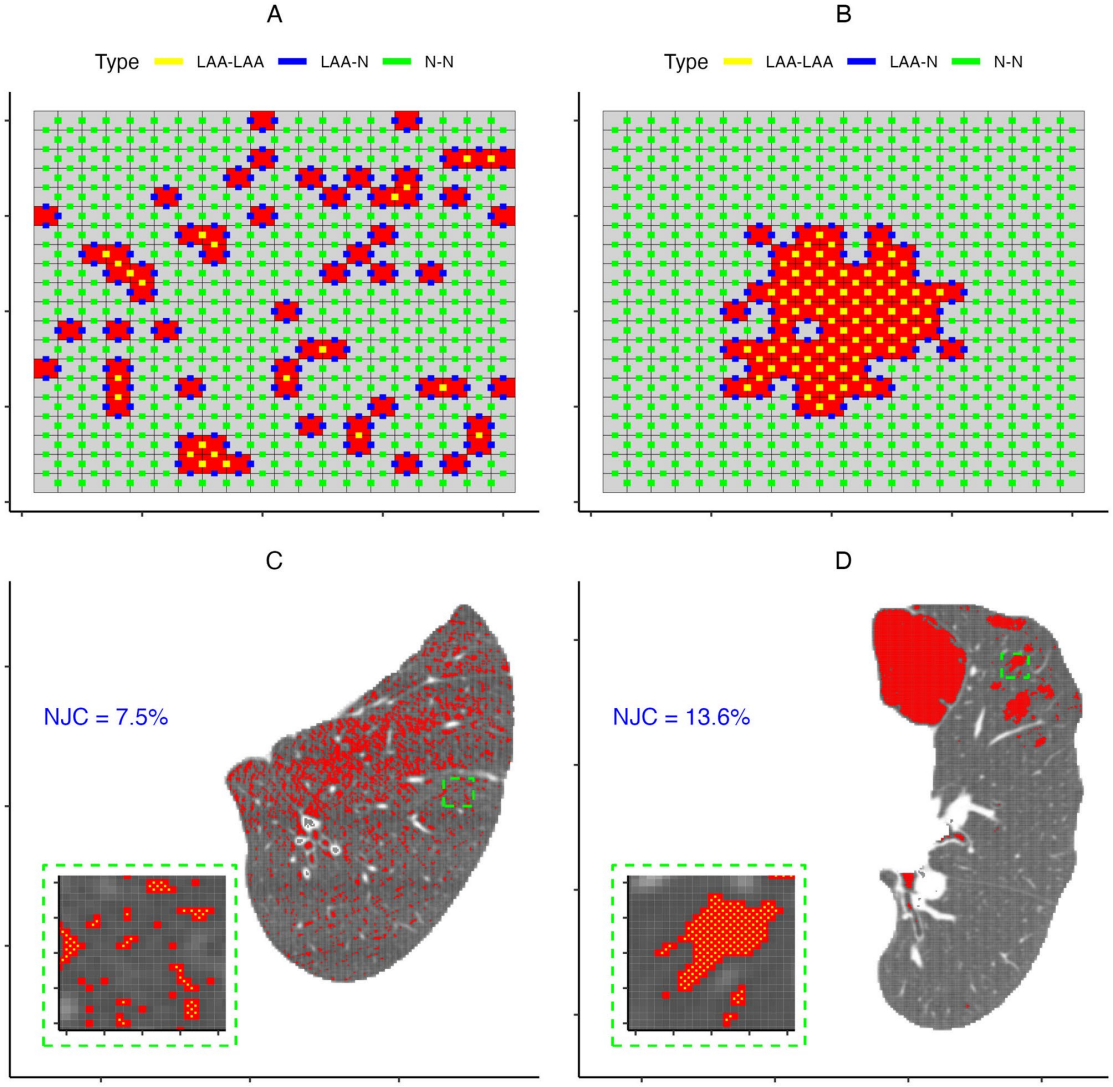
Top Row: Two simulated patterns illustrating how the normalized join counts are calculated where the different types of joins are marked with different colored lines intersecting the shared edges. Both patterns have the same number of LAA voxels (red), but one was generated by a homogeneous process (**A**) while the other was generated using a single multivariate normal distribution (**B**), hence the large difference in NJC (3% in A vs 11% in **B**). Bottom Row: Panels (**C**,**D**) have the two example CT slices used in Figs. 1 and 2 where the zoomed in boxes show the the LAA-to-LAA joins in yellow. *LAA* low attenuation area, *N* normal, *NJC* normalized join-count.

The top row of Fig. 3 has two simulated examples of binary maps that illustrate how the NJCs proposed by Virdee et al.^24^ are computed. In these 2D examples, each shared edge between two voxels constitutes a “join”, and there are three possible types: normal-to-normal, normal-to-LAA, and LAA-to-LAA. NJC is calculated as the number of LAA-to-LAA joins divided by the total number of joins across all three types. The bottom row of Fig. 3 shows an application of this to the two example CT slices used in Figs. 1 and 2 where just the LAA-to-LAA joins are denoted by yellow lines intersecting the shared edges between any two neighboring LAA voxels. Within these two slices, we see that the NJC is substantially higher for the one on the right due to the more compact and clustered nature of the LAA voxels compared to the more scattered distribution, and hence lower NJC, in the slice on the left. For the actual analysis, NJC was computed in 3D where joins were determined by the shared faces of voxels.

#### Spatial point process model

The model proposed in Vestal et al.^17^ is a hierarchical Poisson spatial point process where a latent process governs the number and locations of cluster centers, and a set of independent child processes (one associated with each cluster center) determine the spatial distribution of LAA voxels based on a multivariate normal distribution kernel. The clusters here are not required to be contiguous and their size and shape are governed by cluster-specific parameters. Moreover, this model also includes a homogeneous “scatter” or “noise” component that allows the model the flexibility to quantify both clustered and diffuse disease. This piece is similar in spirit to the metric described in Vestal et al.^28^ where the authors demonstrated that the percentage of LAA voxels that did not show evidence of clustering was associated with pulmonary function. However, that value came from a voxel-wise test based on kernel density smoothing, not from a parametric model fit.

**Figure 4:**
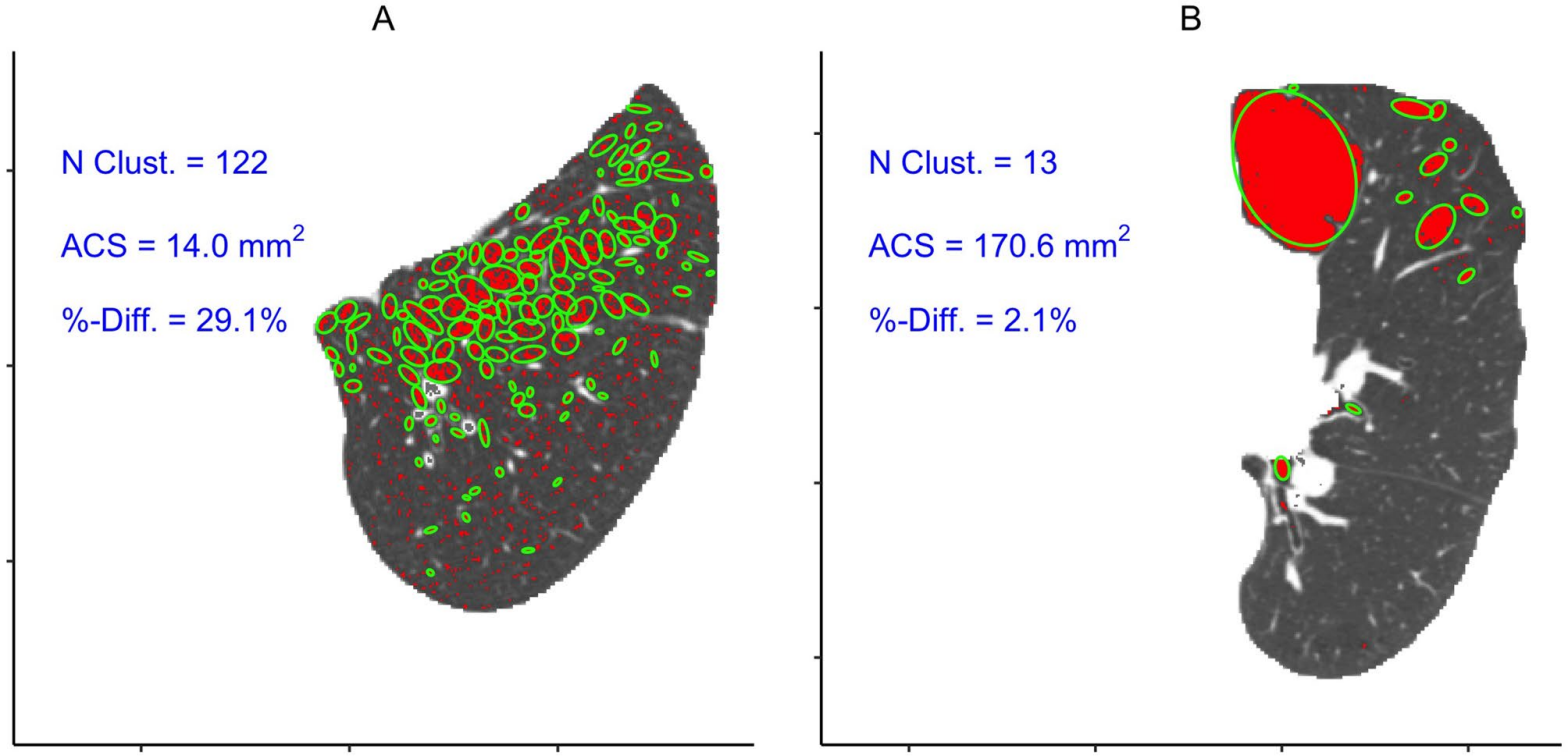
An example application of the spatial point process model to the example slices used in Figs. 1, 2, 3 where approximate cluster boundaries are marked by the green ellipsoids. *ACS* average cluster size.

The results from an example application of the full point process model are shown in Fig. 4. For both slices the spatial point process model estimated far fewer clusters than the connected components strategy used for the power law exponent did, especially in the pattern with more diffuse LAAs in the left panel. In their original paper, Vestal et al (2019) described a Bayesian hierarchical procedure that used spatial Birth-Death Markov Chain Monte Carlo sampling to estimate the relevant point process parameters, and utilities to do so were released as part of the sncp R package^17^. In general, we followed a similar procedure to estimating the clustering parameters as was done in the original paper by analyzing each individual 2D axial slice separately using a Bayesian framework, and then averaging across the slices to obtain subject-level values. Even though the spatial point proccess model easily generalizes to 3D, this strategy was necessary due to computational limitations of the available software as trying to analyze an entire 3D point pattern with potentially millions of LAA voxels would take exponentially longer than analyzing each 2D slice on its own. Only the slices with at least 100 total lung voxels within each scan were analyzed to avoid instability in model estimation around the very top and bottom of the lungs. This model has the flexibility to quantify a large number of features that can describe various aspects of the clustering behavior of LAAs, but we focus on four particular ones that are listed in Table 2: number of clusters (NC; presented in terms of a rate per 100 cm$^2$ of lung tissue), average cluster size (ACS), which was converted from number of voxels to mm$^2$ based on voxel dimensions within a given scan, the amount of voxels that do not show evidence of clustering (%-Diffuse), and average cluster area (ACA), which corresponds to the area covered by the 90$^{th}$ percentile ellipsoids for each cluster (e.g. green features in Fig. 4).

### Visual assessment

Visual assessments of all CT scans in Phase 1 of COPDGene were done based on the 2015 Fleischner Society classification system as previously described in Lynch et al.^29^ and Lynch et al.^15^. In short, each inspiratory CT scan acquired in the COPDGene study was visually assessed by trained analysts. For any scans with substantial differences between the two analysts a final assessment was adjudicated by a trained radiologist. The extent of Centrilobular Emphysema (CLE) was evaluated as absent, trace, mild, moderate, confluent, and advanced destructive. The presence of paraseptal emphysema was assigned as absent, mild, or substantial. We used these two categorical variables for comparing the quantitative measures to visual assessment in the regression models described below as each of these visual assessment domains (CLE and paraseptal) were scored separately.

### Plasma protein array

In the plasma biomarker protein array, 114 candidate biomarkers were measured using a 15-panel assay created by Myriad-RBM (Austin, TX) multiplex technology. In line with the original paper, 16 biomarkers were excluded from further analysis as $>95\%$ of the values fell below the Lower Limit of Quantitation (LLOQ)^27^. Another 17 had $>10\%$ and $<95\%$ of values below the LLOQ, and these were turned into binary present-absent variables. The remaining 81 biomarkers underwent an empirical normal quantile transformation by projecting the ranks onto an inverse normal distribution.

### Descriptive analyses

Pearson linear correlations and Spearman rank correlations were computed between all of the various quantitative emphysema measures. To visualize variability in the profiles generated using just the point process model parameters, we utilized t-Distributed Stochastic Neighbor Embedding (tSNE), which is a non-linear dimension reduction technique^30^. The input variables were centered and scaled versions the four measures from the spatial point process model listed above and (last four rows of Table 2), and then each point (i.e. individual CT scan) was assigned a 2D coordinate based on “similarity” to its neighbors. This was done using the tsne R package with a perplexity of 40 and a maximum of 500 iterations.

### Statistical analyses

All statistical analyses were done using various types of regression models fit in R^31^. Every model presented was adjusted for age, sex, BMI, height, and current smoking status. As well, all image-based emphysema measures (e.g. %LAA, NCJ, ACS, etc.) were natural log transformed due to significant skewness in the observed distributions on their raw scales. Finally, all of the quantitative emphysema measures were centered and scaled so that direct comparisons could be made between the magnitude of regression coefficients. In all models, the clinical outcomes or plasma biomarker abundances always served as the dependent variable, and the quantitative emphysema measures served as the independent covariates.

#### Associations with clinical variables

We first focused on comparing the associations between the emphysema characteristics detailed above and seven measures of pulmonary function, patient quality of life, or evidence of small airway disease: FEV$_1$, FVC, FEV$_1$-FVC ratio, Functional Residual Capacity (FRC), FRC-Total Lung Capacity (TLC) ratio, 6-Minute Walk Distance (6MWD), total St. George’s Respiratory Questionnaire (SGRQ) score, and %-Gas-Trapping (%GT; calculated as the percentage of lung voxels with HU $<-856$ in the paired expiratory CT scans). An initial set of “univariate” regression models were fit where each pairwise combination of clinical outcome and emphysema measure were examined one at a time. For example, seven separate models were fit for FEV$_1$ where each of the CT measures listed in Table 2 were included as the covariate of interest one at a time. From each of these models, the standardized regression coefficient, p-value, and R$^2$ (i.e. the amount of variability explained in the outcome) were recorded. Within this framework we also conducted a sensitivity analysis relating to CT acquisition parameters, specifically slice thickness/spacing. We refit all of these models using just the 562 CT scans that had a voxel height of 0.75 mm to see if results were influenced by including the 25 CT scans that had a voxel height of 0.625 mm.

Subsequently, a second analysis was conducted with a selected subset of the quantitative emphysema measures to understand how they perform when analyzed in combination. Similar to the analyses presented in Virdee et al.^24^, we utilized ridge regression here because of the relatively high levels of correlation between certain quantitative emphysema measures. For each of the seven clinical outcomes, two separate multivariate models were fit. In the first, all of %LAA, D, NJC, and ACS were simultaneously included as covariates, in addition to the demographic characteristics described above. From this, the standardized regression coefficients, their 95% confidence intervals, and p-values from t-tests on them were extracted and compared. In the second model, ACS was dropped as a covariate, and then the adjusted R$^2$ was computed and compared to that from the first model as this gave an estimate for how much additional variability in that clinical measure was explained by adding ACS to a model that already accounted for the other three emphysema measures. In all models, the ridge parameter was estimated using the KKM9 procedure as implemented in the lmridge R package^32^.

In a third analysis, we fit another set of regression models to the clinical outcomes to interrogate how ACS compared to visual assessment. Because we no longer had issues with multicollinearity and we needed to perform multiple degree of freedom tests, we again utilized regular linear regression here instead of ridge regression. Otherwise, the strategy was largely the same where for each outcome a “full” model was fit that included ACS, the two categorical variables describing CLE and paraseptal emphysema respectively, and the standard demographic variables. Next, three reduced models were fit where each of ACS and the two visual assessment variables were dropped individually. Likelihood ratio tests were then conducted between each of these reduced models and the full one, and the p-values were used to compare the strengths of association between either ACS or the two visual assessment components and each clinical variable.

#### Associations with plasma biomarkers

In the original paper, Carolan et al.^27^ demonstrated relationships between numerous markers in this panel and %LAA. To build upon this, we were interested in identifying features that were associated with Emphysema Heterogeneity Phenotypes (EHPs) after accounting for overall burden as measured via %LAA. To do so, we created two EHPs where the first used only NJC and D (EHP2) while the second contained NJC, D, ACS, and the average number of clusters from the point process model (EHP4). We again fit several linear regressions for each biomarker (always the outcome) where first a base model was estimated using just the demographic variables and %LAA as predictors. A second model was fit after adding the EHP2 covariates to the base set, and then the same was done after adding the EHP4 covariates to the base set for a third fit. A likelihood ratio test was conducted between the EHP2 model and the base model to identify features that were associated with that version of an EHP, and then the same was done between the EHP4 model and the base one. Normal linear regression was used for the plasma biomarkers that retained continuous abundance values while logistic regression was used for those that were converted to present/absent based on the preprocessing described above. All p-values were adjusted for multiple comparisons using the Benjamini-Hochberg^33^ method for controlling the False Discovery Rate (FDR), and an FDR threshold of 0.10 was used to determine significance. Differences in the number of biomarkers with significant associations between EHP2 and EHP4 were used to determine if adding the point process measures to NJC and D resulted in increased sensitivity.

## Results

**Figure 5:**
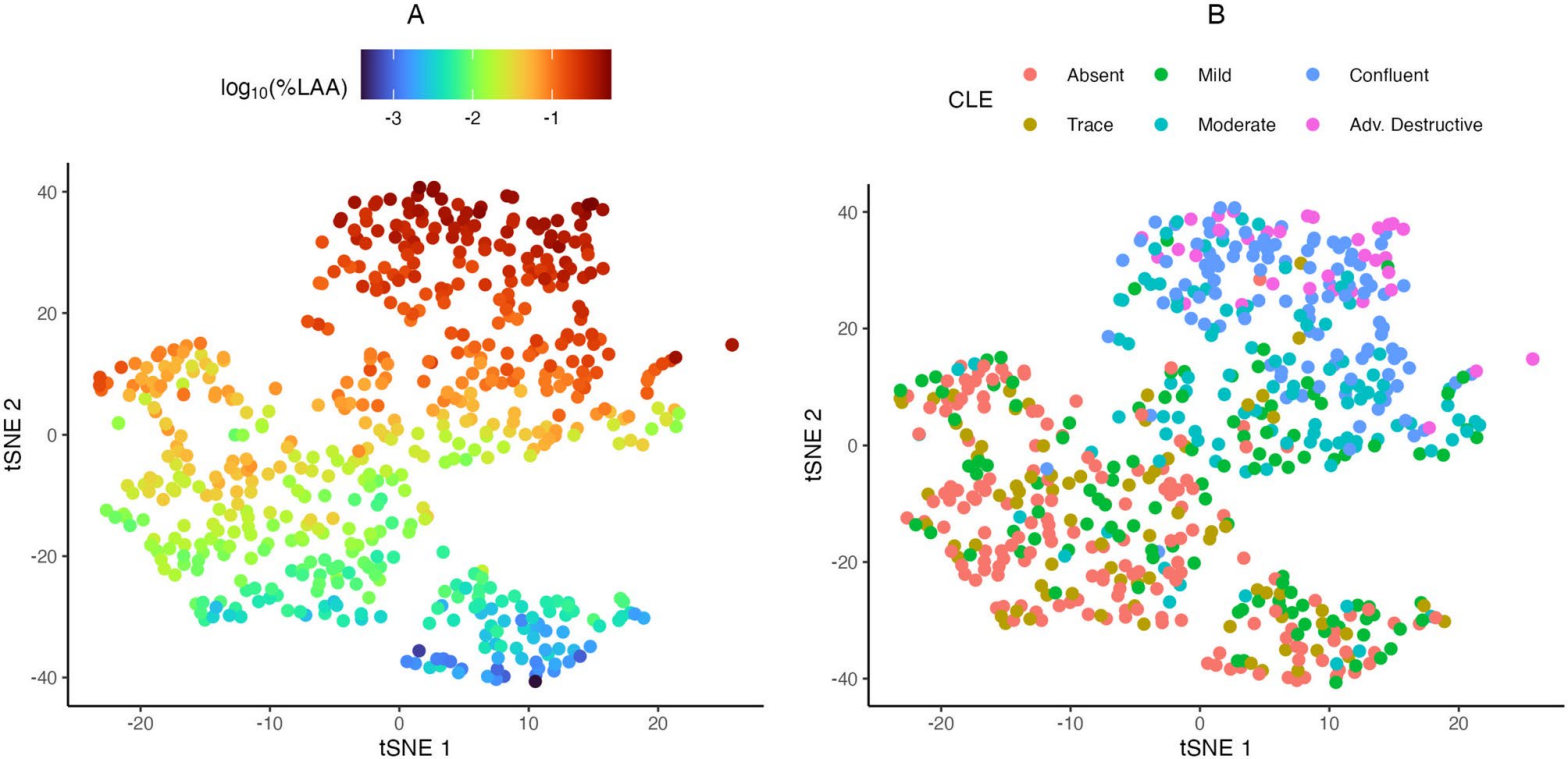
tSNE embeddings of each individual CT scan based on the spatial point process clustering characteristics. The left panel is colored by %LAA on the $\log _{10}$ scale while the right is colored by visual assessment of centrilobular emphysema severity (CLE). *LAA* low attenuation area.

The observed linear correlations between selected quantitative emphysema metrics are shown in Table 3, while the rank-correlations and observed distributions of each individual measure are available in Supplementary Table S1 and Supplementary Figure S1 respectively. As one might expect, there are generally high levels of correlation between most of the measures of emphysema heterogeneity. The two panels of Fig. 5 (and Supplementary Figure S2) show the tSNE embeddings based on the point process model parameters. All of these have the same points, but they are each colored by a different quantitative emphysema metric or visual assessment of CLE. Based on the left panel of Fig. 5, the y-axis (tSNE 2) generally follows the gradient of %LAA where the CTs with low emphysema burden are found towards the bottom and those with high %LAA are all towards the top. However, within scans with similar %LAA values there is substantial variability along the x-axis (tSNE 1), which shows that the spatial clustering measures can resolve different emphysema presentations that would be collapsed if just using %LAA. While the people with advanced destructive and confluent CLE classifications generally group together, there is a large amount of overlap and intermingling of the visual assessment groups suggesting that the emphysema profiles based on the spatial model are not simply recapitulating CLE visual assessment (right panel of Fig. 5 and Supplementary Figure S3). With respect to paraseptal emphysema, we did not find any particularly strong relationships between visually assessed severity and any of the point process measures, which is not unexpected given how little of the overall emphysema burden is likely to be paraseptal in any given CT scan (Supplementary Figure S4).

**Table 3:** Pearson linear correlations between the quantitative emphysema measures.

	%LAA	D	NJC	ACS	NC	%-Diffuse	ACA
%LAA	1.000	− 0.586	0.993	0.877	0.899	− 0.856	0.775
D	− 0.586	1.000	− 0.644	− 0.502	− 0.599	0.769	− 0.112
NJC	0.993	− 0.644	1.000	0.897	0.882	− 0.880	0.730
ACS	0.877	− 0.502	0.897	1.000	0.625	− 0.698	0.758
NC	0.899	− 0.599	0.882	0.625	1.000	− 0.872	0.592
%-Diffuse	− 0.856	0.769	− 0.880	− 0.698	− 0.872	1.000	− 0.484
ACA	0.775	− 0.112	0.730	0.758	0.592	− 0.484	1.000

*LAA* low attenuation area, *NJC* normalized join-count, *ACS* average cluster area, *NC* number of clusters, *ACA* average cluster area.

### Associations with clinical variables

The results from the “univariate” analyses where each combination of clinical outcome and emphysema metric were compared one at a time are presented in Table 4. Here we see that every combination shows highly significant associations with p-values ranging from $10^{-8}$ to $10^{-186}$. However, some patterns start to emerge in terms of ranking the quantitative emphysema measures where NJC has the smallest p-value and largest R$^2$ for each outcome out of it, %LAA, and D. Of the spatial point process measures, ACS is unquestionably the strongest here, and in all cases it has substantially lower p-values and larger R$^2$ than any other measure examined. We also found that voxel height had no impact here as our regression modeling results using just a subset of the scans that all had the same voxel height were nearly identical (see Supplementary Table S2).

**Table 4:** Standardized coefficients, standard errors, *p*-values, and R$^2$ from the “univariate” linear regression models relating all seven quantitative emphysema metrics investigated to each of the seven clinical characteristics of interest.

	Standardized coefficient	S.E.	*p*-value	R$^2$
%GT				
%LAA	0.194	0.005	1.0 x $10^{-147}$	0.778
D	− 0.131	0.009	1.8 x $10^{-37}$	0.441
NJC	0.199	0.005	6.5 x $10^{-158}$	0.796
ACS	0.199	0.004	7.8 x $10^{-186}$	0.839
NC	0.137	0.008	2.6 x $10^{-54}$	0.514
%-Diffuse	− 0.155	0.008	6.6 x $10^{-68}$	0.566
ACA	0.166	0.007	2.5 x $10^{-89}$	0.638
FEV$_1$/FVC				
%LAA	− 0.153	0.006	6.5 x $10^{-96}$	0.585
D	0.133	0.008	2.4 x $10^{-49}$	0.400
NJC	− 0.160	0.006	1.1 x $10^{-106}$	0.619
ACS	− 0.166	0.005	3.7 x $10^{-130}$	0.684
NC	− 0.100	0.007	1.6 x $10^{-35}$	0.330
%-Diffuse	0.132	0.007	3.2 x $10^{-61}$	0.454
ACA	− 0.132	0.007	4.4 x $10^{-63}$	0.462
FEV$_1$				
%LAA	− 0.612	0.033	1.2 x$10^{-59}$	0.542
D	0.574	0.041	3.1 x$10^{-39}$	0.462
NJC	− 0.645	0.033	1.8 x $10^{-66}$	0.566
ACS	− 0.692	0.030	2.2 x $10^{-85}$	0.626
NC	− 0.373	0.038	2.8 x $10^{-21}$	0.380
%-Diffuse	0.502	0.037	3.1 x $10^{-36}$	0.449
ACA	− 0.520	0.036	5.7 x $10^{-40}$	0.465
FRC				
%LAA	0.735	0.039	4.7 x $10^{-62}$	0.590
D	− 0.530	0.051	2.2 x $10^{-23}$	0.434
NJC	0.764	0.038	2.6 x $10^{-67}$	0.608
ACS	0.803	0.034	1.6 x $10^{-84}$	0.660
NC	0.492	0.045	2.8 x $10^{-25}$	0.443
%-Diffuse	− 0.585	0.045	2.8 x $10^{-34}$	0.483
ACA	0.639	0.042	1.9 x $10^{-44}$	0.525
FRC/TLC				
%LAA	0.061	0.005	4.3 x $10^{-35}$	0.337
D	− 0.054	0.005	3.4 x $10^{-21}$	0.256
NJC	0.064	0.005	2.1 x $10^{-38}$	0.355
ACS	0.071	0.004	1.1 x $10^{-52}$	0.428
NC	0.037	0.005	1.2 x $10^{-12}$	0.202
%-Diffuse	− 0.051	0.005	6.5 x $10^{-23}$	0.267
ACA	0.055	0.005	1.8 x $10^{-27}$	0.294
SGRQ (Total)				
%LAA	13.276	0.953	2.7 x $10^{-38}$	0.261
D	− 13.651	1.095	9.5 x $10^{-32}$	0.222
NJC	14.078	0.944	7.8 x $10^{-43}$	0.287
ACS	15.444	0.873	2.4 x $10^{-56}$	0.359
NC	7.148	1.032	1.1 x $10^{-11}$	0.088
%-Diffuse	− 11.039	1.020	5.6 x $10^{-25}$	0.179
ACA	11.642	0.997	2.0 x $10^{-28}$	0.201
6MWD				
%LAA	− 169.800	16.441	6.0 x $10^{-23}$	0.243
D	166.934	18.829	1.1 x $10^{-17}$	0.209
NJC	− 180.141	16.477	2.8 x $10^{-25}$	0.258
ACS	− 211.532	15.917	4.3 x $10^{-35}$	0.317
NC	− 92.191	16.845	6.8 x $10^{-08}$	0.142
%-Diffuse	137.284	17.187	8.3 x $10^{-15}$	0.190
ACA	− 150.377	17.078	1.8 x $10^{-17}$	0.208

*LAA* low attenuation area, *NJC* normalized join-count, *ACS* average cluster size, *NC* number of cluster, *ACA* average cluster area, *FEV*$_1$ forced expiratory volume in one second, *FVC* forced vital capacity, *6MWD* 6-minute walk distance, *FRC* functional residual capacity, *TLC* total lung capacity, *GT* gas trapping.

**Figure 6:**
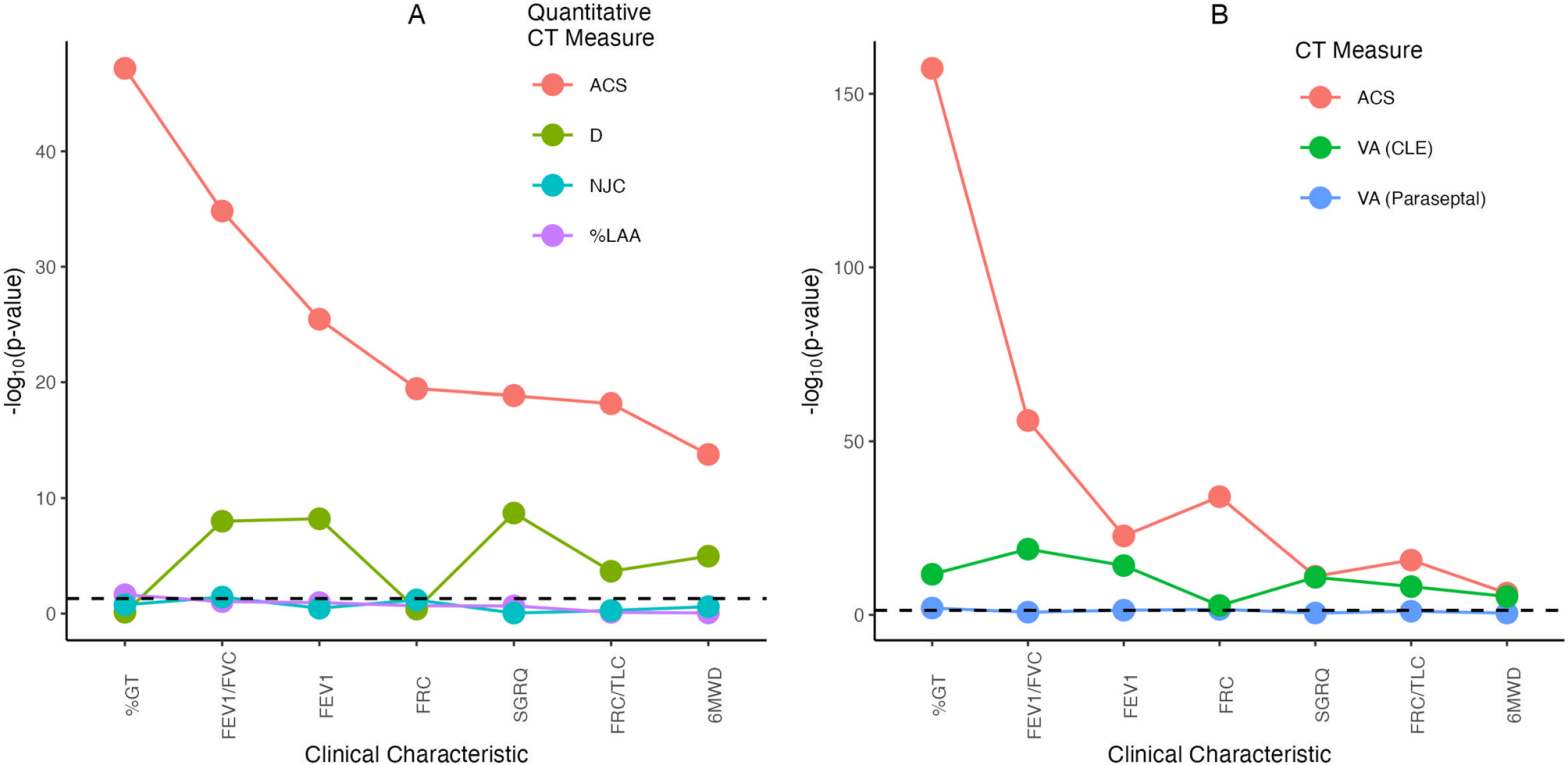
Panel (**A**) shows the *p*-values, on the $-\log _{10}$ scale, for each combination of quantitative emphysema metric and clinical characteristic based on the ridge regression results. Panel (**B**) shows the *p*-values for likelihood ratio tests for either ACS, visual assessment (VA) of centrilobular emphysema (CLE), or visual assessment of paraseptal emphysema from the linear regression models fit to each clinical outcome. The horizontal dashed line represents $p=0.05$ in both panels. *LAA* low attenuation area, *NJC* normalized join-count, *ACS* average cluster size, *FEV*$_1$ forced expiratory volume in one second, *FVC* forced vital capacity, *6MWD* 6-minute walk distance, *FRC* functional residual capacity, *TLC* total lung capacity, *GT* gas trapping.

**Figure 7:**
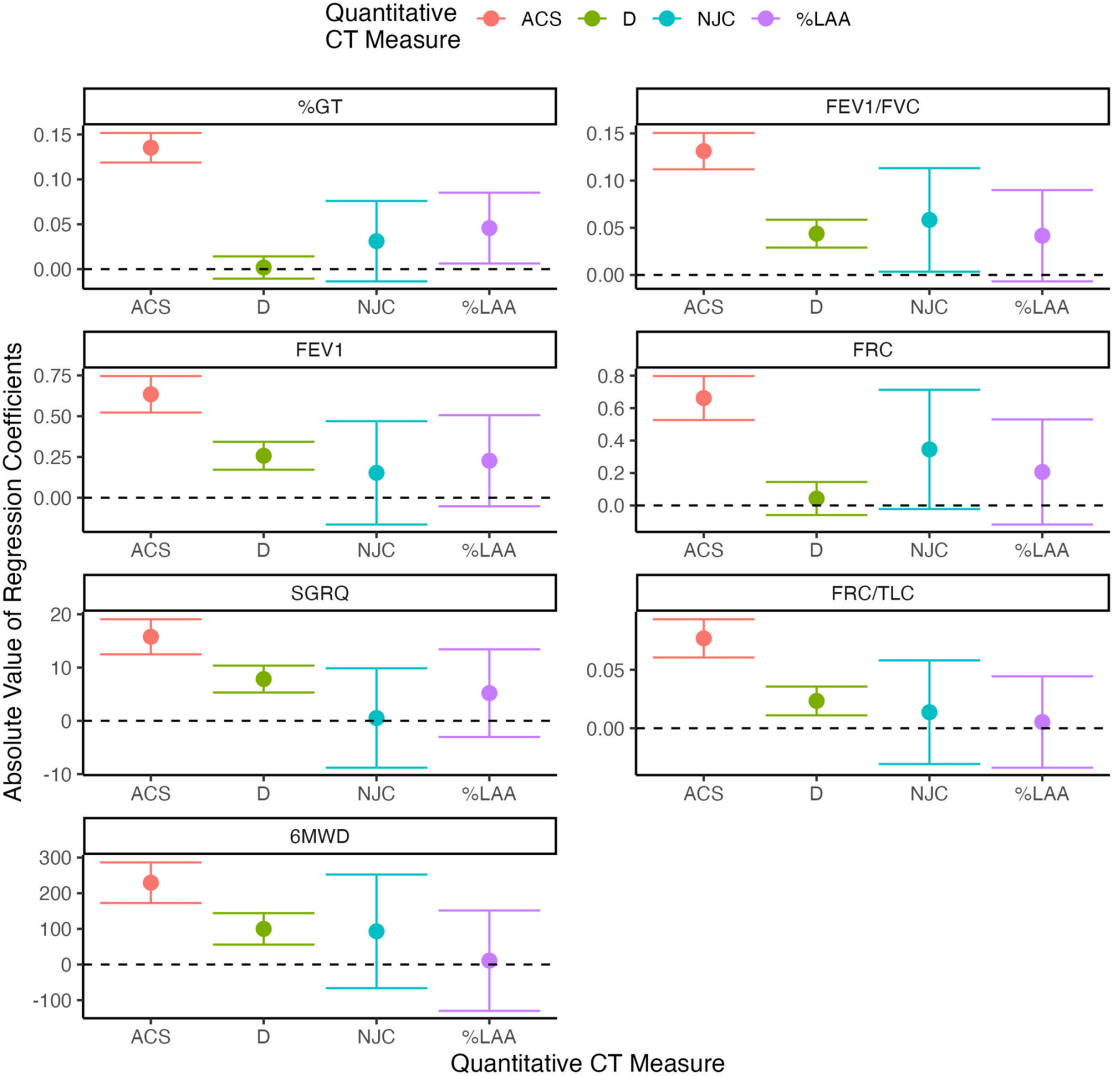
Absolute value of the point estimates and 95% confidence intervals for the regression coefficients from each combination of quantitative emphysema measure and clinical outcomes of interest. Note that the quantitative emphysema measures were all mean-centered and scaled by their standard deviations, so the values represent the absolute change in the outcome for every standard deviation increase in that measure. *LAA* low attenuation area, *NJC* normalized join-count, *ACS* average cluster size, *FEV*$_1$ forced expiratory volume in one second, *FVC* forced vital capacity, *6MWD* 6-minute walk distance, *FRC* functional residual capacity, *TLC* total lung capacity, *GT* gas trapping.

Results from the ridge regression models that simultaneously related %LAA, D, NJC and ACS to the seven clinical variables of interest can be found in Table 5 with visualizations of the regression p-values and coefficients shown in Figs. 6 and 7 respectively. We again found that ACS showed the strongest associations for all the outcomes with p-values many orders of magnitude smaller than what was seen for any other variable of interest. This was also observed for the standardized coefficients as the values for ACS were at least about twice as large in absolute value as those for NJC, D or %LAA. Both NJC and %LAA seemed to be redundant and add little information after adjusting for both ACS and D in these models. Although the p-values and standardized coefficients for D are nowhere near as strong as those for ACS, they are still quite significant for five of the seven outcomes, which suggests D and ACS do contain complimentary information. Table 6 compares adjusted R$^2$2 values for models that contain all of ACS, D, NJC, and %LAA and a set of reduced models that only contains the latter three. A substantial increase in adjusted R$^2$ was noted when ACS is included with relative improvements between 8%-27%.

**Table 5:** Standardized coefficients, standard errors, and p-values from the multivariable ridge regression models simultaneously relating the top four quantitative emphysema metrics investigated to each of the seven clinical characteristics of interest.

	Standardized coefficient	S.E.	*p*-value
FEV$_1$/FVC			
ACS	− 0.14	0.01	2.4 x $10^{-31}$
D	0.04	0.01	7.3 x $10^{-08}$
NJC	− 0.08	0.03	0.005
%LAA	0.08	0.03	0.003
FEV $_1$			
ACS	− 0.68	0.07	1.5 x $10^{-23}$
D	0.25	0.04	2.7 x $10^{-08}$
NJC	− 0.25	0.16	0.129
%LAA	0.38	0.14	0.008
FRC			
ACS	0.76	0.08	2.2 x $10^{-20}$
D	− 0.04	0.05	0.439
NJC	0.37	0.19	0.053
%LAA	− 0.34	0.17	0.044
FRC/TLC			
ACS	0.09	0.01	3.2 x $10^{-18}$
D	− 0.02	0.01	3.3 x $10^{-04}$
NJC	− 0.01	0.02	0.753
%LAA	− 0.02	0.02	0.275
SGRQ (Total)			
ACS	17.00	1.92	1.2 x $10^{-17}$
D	− 7.60	1.29	7.2 x $10^{-09}$
NJC	3.01	4.86	0.535
%LAA	− 9.14	4.26	0.032
6MWD			
ACS	− 249.28	33.34	3.1 x $10^{-13}$
D	96.46	22.59	2.3 x $10^{-05}$
NJC	58.51	82.32	0.478
%LAA	47.49	72.30	0.512

*LAA* low attenuation area, *NJC* normalized join-count, *ACS* average cluster size, *FEV*$_1$ forced expiratory volume in one second, *FVC* forced vital capacity, *6MWD* 6-minute walk distance, *FRC* functional residual capacity, *TLC* total lung capacity, *GT* gas trapping.

When comparing to visual assessment, we also found ACS to be highly significant in every model (right panel of Fig. 6). For each outcome besides FRC, visual assessment of CLE also had very significant LRT p-values. ACS had p-values multiple orders of magnitude smaller than visual assessment for FEV$_1$, FEV$_1$-FVC ratio, FRC, and FRC-TLC ratio. However, for both 6MWD and SGRQ score, the p-values were essentially the same for both ACS and visual assessment of CLE. This suggests that even though ACS drastically outperformed existing quantitative metrics and visual assessment of CLE in every head-to-head comparison, there is still substantial complementary information in visual assessment that helps explain differences in pulmonary function between individuals. After accounting for both ACS and visual assessment of CLE, visual assessment of paraseptal emphysema was not significantly associated with any of the outcomes.

### Associations with plasma biomarkers

The entire set of plasma biomarkers that had an FDR $<0.10$ for either of the two likelihood ratio tests are presented in Supplementary Table S3. Overall, 17 of these were found to have a significant association with EHP2, while 30 (76% increase) were found when using the expanded EHP4. Of the 31 proteins identified using either model, 16 were found using both EHPs, only one was found to have a significant association EHP2 but not with EHP4, and 14 (1300% increase) were found using the whole set of imaging variables present in EHP4 but not when using just EHP2. This latter set is detailed in Table 7 and includes the advanced glycosylation end-product specific receptor (AGER) gene that has been shown to have significant associations with COPD, emphysema, and %LAA at genetic, genomic, and proteomic levels^27,34–36^.

**Table 6:** Adjusted R$^2$ values from the ridge regression models that included all of %LAA, NJC, D, and ACS (Full) or just the former three (Red.).

Outcome	Adj. R$^2$ (Red.)	Adj. R$^2$ (Full)
FEV$_1$	0.576	0.646
SGRQ(Total)	0.310	0.395
6MWD	0.267	0.334
FEV$_1$/FVC	0.624	0.709
%GT	0.793	0.855
FRC	0.606	0.660
FRC/TLC	0.358	0.436

*LAA* low attenuation area, *NJC* normalized join-count, *ACS* average cluster size, *FEV*$_1$ forced expiratory volume in one second, *FVC* forced vital capacity, *6MWD* 6-minute walk distance, *FRC* functional residual capacity, *TLC* total lung capacity, *GT* gas trapping.

**Table 7:** Genes with an FDR $<0.10$ for an association between abundance and EHP4 that had an FDR $>0.10$ for an association with EHP2, both after accounting for overall emphysema burden with %LAA, age, sex, height, BMI, and current smoking status.

Gene	FDR with EHP4	FDR with EHP2
AGER	0.001	0.175
CXCL9	0.006	0.114
ADIPOQ	0.011	0.114
CXCL10	0.011	0.332
HP	0.017	0.332
SOD1	0.019	0.239
CCL11(P/A)	0.021	0.175
MB	0.036	0.114
SERPINA1	0.074	0.227
MICA (P/A)	0.074	0.143
CCL11	0.084	0.197
CCL20	0.084	0.296
CCL5	0.085	0.114
CSTB	0.093	0.136

*P/A* continuous abundance was converted into present/absent based on the preprocessing, *LAA* low attenuation area, *EHP* emphysema heterogeneity phenotype.

## Discussion

In this work, we have shown that summarizing the clustering characteristics of radiologically based emphysema present in a chest CT scan using a spatial point process framework gives significantly stronger associations with both clinically relevant outcomes and plasma protein abundances than using other existing methods. Even though they are more computationally expensive to compute, the clustering measures have the benefit of simple physical interpretations with respect to the disease process compared to alternatives like the power law exponent D and NJC: number of clusters (lesions), average size or area of the clusters, and the proportion of diseased tissue that did not cluster. Taken together, the collection of spatial clustering measures can separate distinct patterns/presentations that are collapsed when using just %LAA values, and the most informative univariate measure (ACS) vastly outperforms every alternative quantification of emphysema heterogeneity we compared to.

Our results generally align with the findings of Mishima et al.^19^ and several subsequent follow-ups^20–23^. They found smaller values of the power law exponent in patients with COPD than in normal controls, which implies a shift towards larger LACs in the size distribution and a corresponding loss of complexity in the tissue overall. They suggested that the size of an LAC is related to local blood-gas exchange characteristics, and that for a given %LAA, numerous small clusters give a larger surface area for gas exchange than fewer larger clusters do. The more complex spatial model we used here generates results that agree with this hypothesis: larger ACS was uniformly associated with worse pulmonary function. Moreover, when they were compared directly, the ACS metric greatly outperformed the estimated fractal dimension D. This could be a result of the fact that the spatial point process model relaxes the definition of a cluster away from connected components and that it allows for both diffuse and clustered disease while the power law estimation method includes all LAA voxels in the clustering process where even singletons are treated as “clusters”. Even so, D was still found to be significant, albeit at much lower levels than ACS, for six of the seven clinical characteristics, and thus it does seem to encode some complimentary information that ACS alone does not capture.

We also found ACS to have noticeably stronger associations with five of the seven outcomes explored than the combination of the two visual assessment variables, while for the other two outcomes the p-values for ACS were essentially equivalent to assessment of CLE. In all models, visual assessment of paraseptal emphysema did not have a significant association with the outcomes after accounting for ACS and CLE. The spatial point process model was motivated to quantify some aspects of visual assessment (i.e., separating centrilobular and panlobular emphysema presentations and evaluating the severity of both), but there is still a significant amount of relevant information in the visual assessments of CLE that the model is seemingly not able to capture. This is consistent with results from other studies that have found visual assessment and %LAA contribute independent information (e.g. Kirby et al.^18^ and Lynch et al.^15^). This could be in part because the visual scoring was focused on the identifying the most severe pattern observed in each CT, not the most prevalent. Alternatively, our measures are taken as means over the entirety of the lungs, so they are more indicative of average presentation. Even though ACS greatly outperformed %LAA, NJC and D, it can still be seen as complimentary to visual assessment of CLE (and vice versa), and thus the most comprehensive emphysema profiling should contain both aspects.

For the plasma protein expression levels, generating an enhanced EHP by adding in ACS and the average number of clusters from the point process model to D and NJC resulted in many more discoveries overall and more unique associations. These results, in conjunction with the stronger associations with pulmonary function, suggest these markers are substantially more powerful than the alternatives in a cross-sectional setting. A next major step in the development of these point process based imaging biomarkers is to establish their behavior longitudinally where one can explore how changes in the spatial measures relate to changes in, among others, pulmonary function, exacerbations, and mortality.

One limitation of this study is the low representation of GOLD stage 1 patients in this cohort. This early disease stage group is an important cohort of individuals for clinical trials and understanding disease progression. In future studies, we plan to expand our application of the spatial point process model to more of the COPDGene patient population which will allow us to have better representation of this group of individuals. Another limitation regarding the spatial point process modeling is that the current software implementation is limited to only analyzing 2D slices, and thus a full 3D characterization of LAA clusters is not currently possible. This is strictly a computational limitation as the mathematical model easily generalizes to 3D, but the number of LAA voxels involved in analyzing an entire lung in 3D with even moderate %LAA (e.g. $\approx 10\%$) would likely be around 1-2 million. This is orders of magnitude higher than what is analyzed using the 2D strategy as each individual axial slice would contain closer to just a few thousand, making the model fits much more manageable. While this means that the subject-level clustering summaries do not yet characterize all available spatial data and should in theory be at a disadvantage to the other measures that were computed in 3D, our regression results suggest even the simplified measures calculated as average behavior across the 2D axial slices are already much more informative than existing 3D alternatives.

Ultimately, we have demonstrated that there is significant information related to emphysema distribution encoded in lung CT scans above and beyond what is captured using just %LAA that is relevant to pulmonary function and patient quality of life. Of the available methods that attempt to quantify some aspect of spatial heterogeneity of emphysema distribution, the spatial clustering characteristics originally developed by Vestal et al.^17^ and further explored here were the strongest. However, our results also suggest that a combination of ACS from the point process model and the power law exponent D generate the strongest quantitative emphysema phenotype and show the potential to be powerful imaging biomarkers. In future work, we aim to establish both genetic and genomic associations with these new imaging metrics, and to examine their ability to describe disease progression, where we expect changes in ACS within a subject to be associated with worsening pulmonary function, by leveraging the longitudinal follow-up scans from these same individuals in the later phases of COPDGene.

### Supplementary Information

Supplementary Information.

## Data Availability

The data that support the findings of this study are available from COPDGene but restrictions apply to the availability of these data, which were used under license for the current study, and so are not publicly available. Data are however available from the authors upon reasonable request and with permission of COPDGene. Requests should be directed to the COPDGene Ancillary Studies Committee via Shandi Watts (WattsS@NJHealth.org). Example code showing how to estimate D, NJC, and the spatial point process measures in R using simulated data is available at https://github.com/stop-pre16/Emphysema-quantification-example/.
