# Supplementary material for: Quantifying the spatial clustering characteristics of radiographic emphysema explains variability in pulmonary function
Source: Sci Rep. 2023 Aug 24;13:13862. doi: 10.1038/s41598-023-40950-8 (PMC10449810; doi:10.1038/s41598-023-40950-8)
Supplement: Supplementary file 1 — Supplementary Information. [file 41598_2023_40950_MOESM1_ESM.pdf]

# Quantifying the spatial clustering characteristics of radiographic emphysema explains variability in pulmonary function
# Online Data Supplement

Brian E. Vestal, Debashis Ghosh, Raúl San José Estépar, Katerina Kechris, Tasha Fingerlin, Nichole E. Carlson

# 1 Supplementary Figures and Tables

## 1.1 Figures

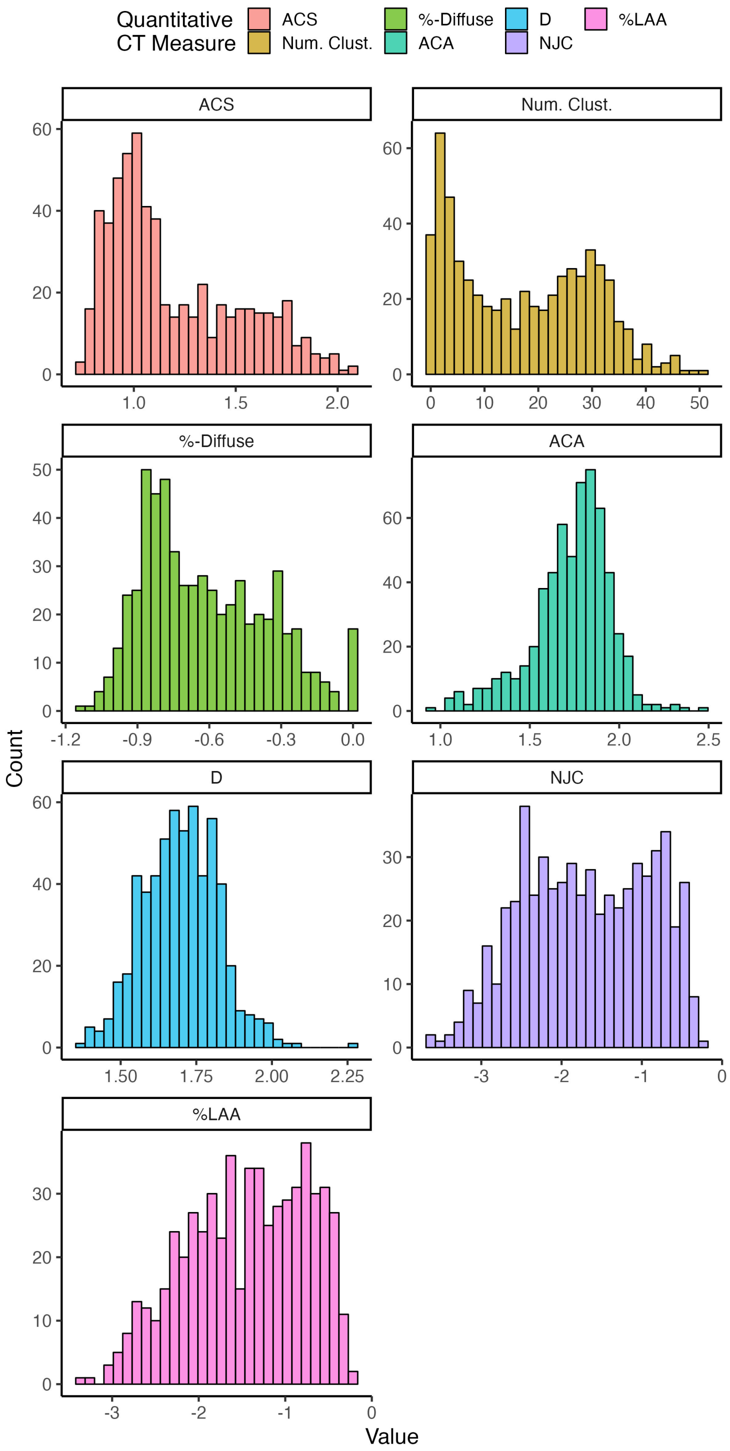


Figure S1: Histograms showing the distributions of quantitative emphysema metric across the 587 CT scans analyzed. ACS and ACA are presented on the natural log scale, while %-Diffuse, NJC, and %LAA are presented on the $\log_{10}$ scale.

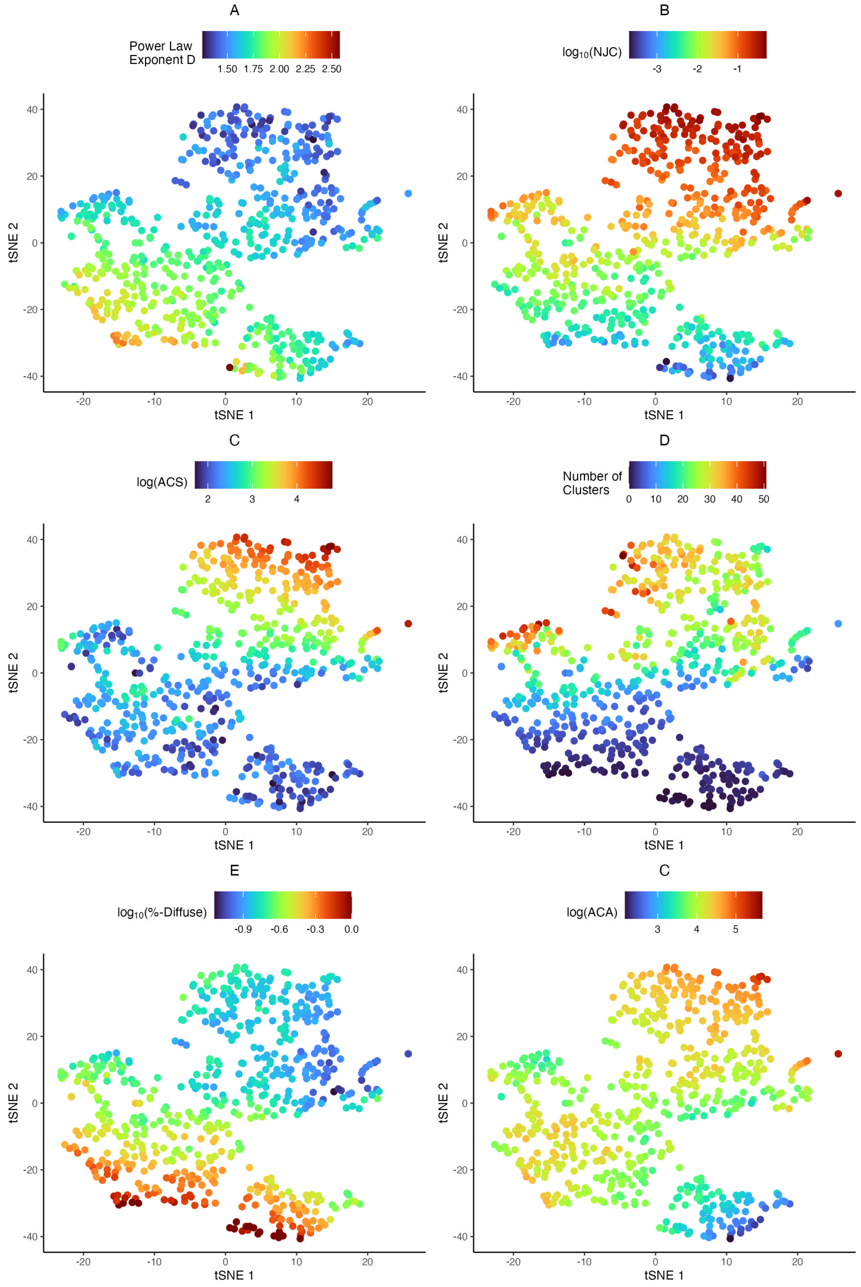


Figure S2: tSNE embeddings of each individual CT scan based on the spatial point process clustering characteristics. Each panel is colored by a different quantitative emphysema metric.

Figure S3: Boxplots showing the distributions of average cluster size (ACS), number of clusters, %-diffuse, and average cluster area (ACA) within each centrilobular emphysema (CLE) visual assessment (VA) category.

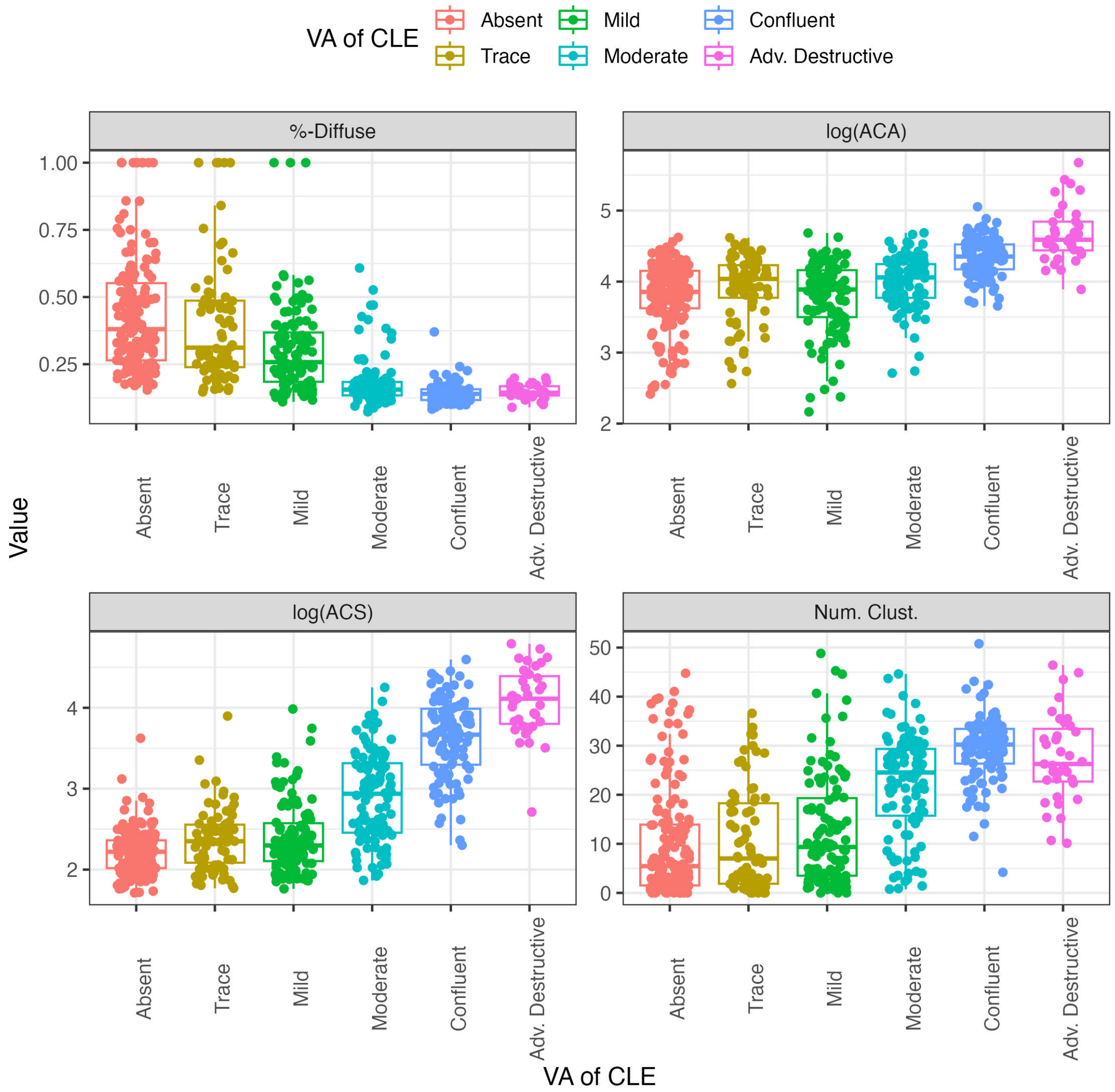

Figure S4: Boxplots showing the distributions of average cluster size (ACS), number of clusters, %-diffuse, and average cluster area (ACA) within each paraseptal emphysema (PE) visual assessment (VA) category.

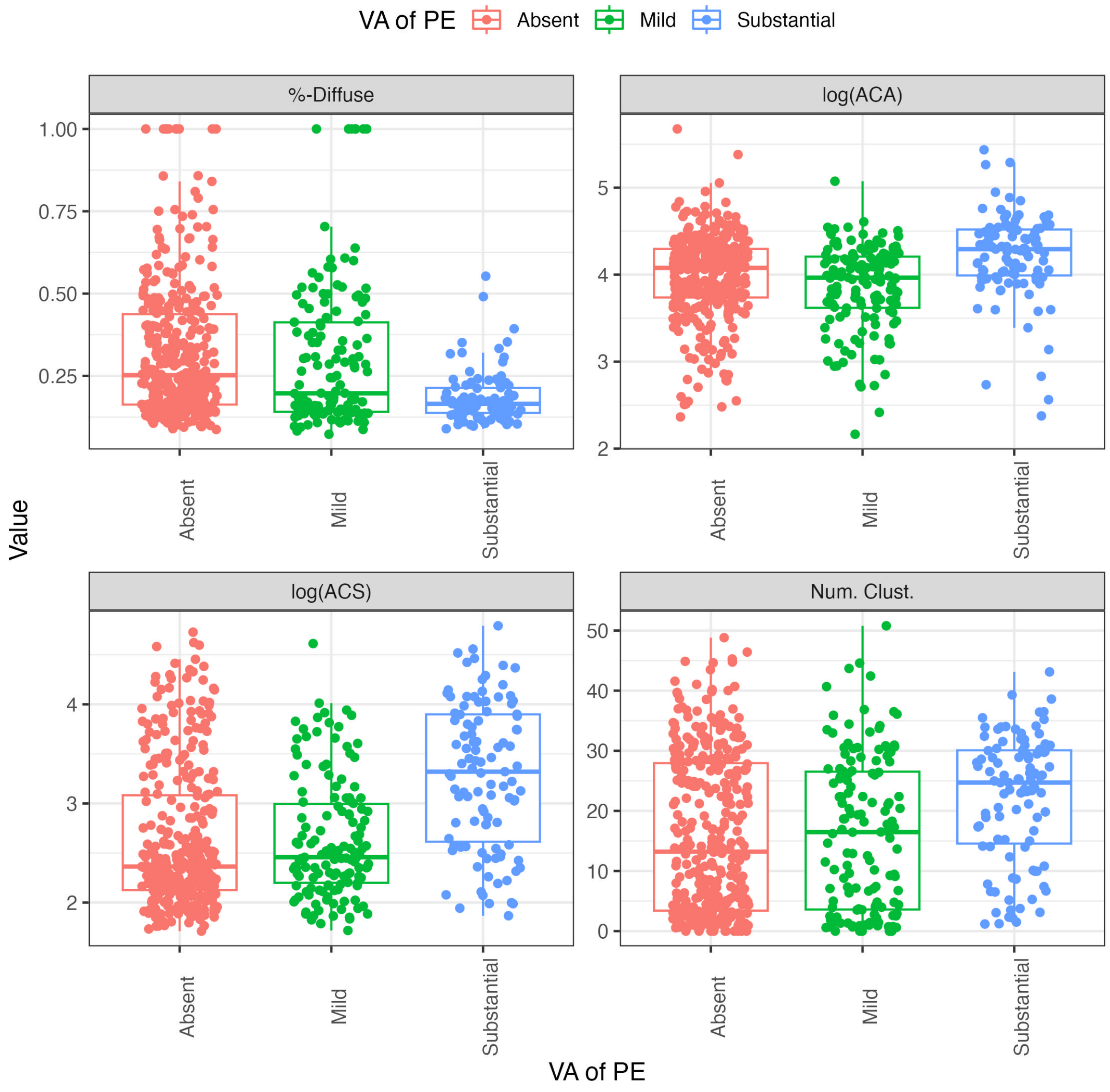

## 1.2 Tables

Table S1: Spearman rank correlations between the quantitative emphysema measures. LAA = low attenuation area, NJC = normalized join-count, ACS = average cluster area, NC = number of clusters, ACA = average cluster area.

| | %LAA | D | NJC | ACS | NC | %-Diffuse | ACA |
|---|---|---|---|---|---|---|---|
| %LAA | 1.000 | -0.608 | 0.996 | 0.923 | 0.907 | -0.843 | 0.753 |
| D | -0.608 | 1.000 | -0.651 | -0.493 | -0.630 | 0.782 | -0.115 |
| NJC | 0.996 | -0.651 | 1.000 | 0.919 | 0.902 | -0.870 | 0.720 |
| ACS | 0.923 | -0.493 | 0.919 | 1.000 | 0.756 | -0.780 | 0.821 |
| NC | 0.907 | -0.630 | 0.902 | 0.756 | 1.000 | -0.805 | 0.511 |
| %-Diffuse | -0.843 | 0.782 | -0.870 | -0.780 | -0.805 | 1.000 | -0.463 |
| ACA | 0.753 | -0.115 | 0.720 | 0.821 | 0.511 | -0.463 | 1.000 |

Table S2: Standardized coefficients, standard errors, p-values, and $R^2$ from the "univariate" linear regression models relating all seven quantitative emphysema metrics investigated to each of the seven clinical characteristics of interest using only the subjects with CT scans that had a voxel height of 0.75 mm (n=562)

| | Standardized Coefficient | S.E. | p-value | $R^2$ |
|---|---|---|---|---|
| **%GT** | | | | |
| %LAA | 0.196 | 0.005 | $2.0 \times 10^{-144}$ | 0.785 |
| D | -0.139 | 0.010 | $9.8 \times 10^{-39}$ | 0.458 |
| NJC | 0.201 | 0.005 | $2.7 \times 10^{-156}$ | 0.806 |
| ACS | 0.202 | 0.004 | $4.0 \times 10^{-182}$ | 0.846 |
| NC | 0.138 | 0.008 | $8.8 \times 10^{-53}$ | 0.520 |
| %-Diffuse | -0.157 | 0.008 | $4.2 \times 10^{-66}$ | 0.573 |
| ACA | 0.168 | 0.007 | $9.5 \times 10^{-87}$ | 0.644 |
| **$FEV_1$/FVC** | | | | |
| %LAA | -0.154 | 0.006 | $3.4 \times 10^{-92}$ | 0.589 |
| D | 0.142 | 0.008 | $2.5 \times 10^{-52}$ | 0.428 |
| NJC | -0.161 | 0.006 | $1.9 \times 10^{-103}$ | 0.625 |
| ACS | -0.168 | 0.005 | $1.7 \times 10^{-126}$ | 0.690 |
| NC | -0.100 | 0.008 | $1.2 \times 10^{-33}$ | 0.332 |
| %-Diffuse | 0.133 | 0.007 | $1.1 \times 10^{-59}$ | 0.462 |
| ACA | -0.132 | 0.007 | $7.2 \times 10^{-60}$ | 0.462 |
| **$FEV_1$** | | | | |
| %LAA | -0.614 | 0.034 | $3.9 \times 10^{-57}$ | 0.538 |
| D | 0.613 | 0.042 | $2.5 \times 10^{-41}$ | 0.473 |
| NJC | -0.649 | 0.034 | $3.6 \times 10^{-64}$ | 0.564 |
| ACS | -0.698 | 0.030 | $5.7 \times 10^{-83}$ | 0.627 |
| NC | -0.368 | 0.039 | $9.2 \times 10^{-20}$ | 0.371 |
| %-Diffuse | 0.503 | 0.038 | $1.5 \times 10^{-34}$ | 0.443 |
| ACA | -0.520 | 0.037 | $8.1 \times 10^{-38}$ | 0.458 |
| **FRC** | | | | |
| %LAA | 0.737 | 0.040 | $3.6 \times 10^{-59}$ | 0.596 |
| D | -0.558 | 0.053 | $1.7 \times 10^{-23}$ | 0.449 |
| NJC | 0.768 | 0.039 | $1.5 \times 10^{-64}$ | 0.615 |
| ACS | 0.809 | 0.035 | $4.2 \times 10^{-81}$ | 0.667 |
| NC | 0.489 | 0.046 | $5.8 \times 10^{-24}$ | 0.451 |
| %-Diffuse | -0.589 | 0.046 | $7.2 \times 10^{-33}$ | 0.492 |
| ACA | 0.644 | 0.043 | $1.5 \times 10^{-42}$ | 0.533 |
| **FRC/TLC** | | | | |
| %LAA | 0.062 | 0.005 | $1.8 \times 10^{-36}$ | 0.364 |
| D | -0.060 | 0.005 | $2.4 \times 10^{-25}$ | 0.300 |
| NJC | 0.066 | 0.005 | $2.0 \times 10^{-40}$ | 0.386 |
| ACS | 0.072 | 0.004 | $7.2 \times 10^{-54}$ | 0.454 |
| NC | 0.038 | 0.005 | $1.9 \times 10^{-13}$ | 0.223 |
| %-Diffuse | -0.054 | 0.005 | $1.1 \times 10^{-24}$ | 0.295 |
| ACA | 0.054 | 0.005 | $2.8 \times 10^{-26}$ | 0.305 |
| **SGRQ (Total)** | | | | |
| %LAA | 13.245 | 0.981 | $3.7 \times 10^{-36}$ | 0.255 |
| D | -14.529 | 1.138 | $6.3 \times 10^{-33}$ | 0.235 |
| NJC | 14.077 | 0.971 | $1.3 \times 10^{-40}$ | 0.282 |
| ACS | 15.549 | 0.897 | $4.1 \times 10^{-54}$ | 0.358 |
| NC | 6.946 | 1.061 | $1.4 \times 10^{-10}$ | 0.082 |
| %-Diffuse | -11.058 | 1.051 | $1.0 \times 10^{-23}$ | 0.175 |
| ACA | 11.762 | 1.027 | $2.0 \times 10^{-27}$ | 0.200 |
| **6MWD** | | | | |
| %LAA | -171.842 | 16.833 | $2.1 \times 10^{-22}$ | 0.246 |
| D | 184.454 | 19.587 | $1.5 \times 10^{-19}$ | 0.227 |
| NJC | -182.436 | 16.864 | $1.0 \times 10^{-24}$ | 0.261 |
| ACS | -210.134 | 16.398 | $7.7 \times 10^{-33}$ | 0.312 |
| NC | -96.495 | 17.242 | $3.6 \times 10^{-08}$ | 0.146 |
| %-Diffuse | 140.536 | 17.649 | $1.1 \times 10^{-14}$ | 0.193 |
| ACA | -153.093 | 17.570 | $4.0 \times 10^{-17}$ | 0.210 |

Table S3: All genes with and FDR< .10 for the association between protein abundance and either of the Emphysema Heterogeneity Phenotypes (EHPs) using either just NJC and $D$ (EHP2, right column), or both of those variables and ACS and average number of clusters from the spatial point process model model (EHP4, left column).P/A= continuous abundance was converted into present/absent based on the preprocessing.

| Gene | FDR with EHP4 | FDR with EHP2 |
|---|---|---|
| ICAM1 | 0.001 | 0.001 |
| IL8 | 0.001 | 0.003 |
| MMP9 | 0.001 | 0.002 |
| AGER | 0.001 | 0.175 |
| BDNF | 0.006 | 0.016 |
| CCL13 | 0.006 | 0.004 |
| CXCL9 | 0.006 | 0.114 |
| TIMP2 | 0.006 | 0.003 |
| IL2RA | 0.006 | 0.002 |
| TGFB1-LAP | 0.009 | 0.020 |
| IL1RN (P/A) | 0.009 | 0.051 |
| ADIPOQ | 0.011 | 0.114 |
| CXCL10 | 0.011 | 0.332 |
| CRP | 0.012 | 0.004 |
| CCL18 | 0.012 | 0.014 |
| SFTPD | 0.014 | 0.007 |
| HP | 0.017 | 0.332 |
| SOD1 | 0.019 | 0.239 |
| CCL11 (P/A) | 0.021 | 0.175 |
| CCL8 | 0.024 | 0.051 |
| CCL23 | 0.033 | 0.016 |
| MB | 0.036 | 0.114 |
| SERPINA1 | 0.074 | 0.227 |
| CXCL5 | 0.074 | 0.056 |
| MICA (P/A) | 0.074 | 0.143 |
| CCL11 | 0.084 | 0.197 |
| CCL20 | 0.084 | 0.296 |
| CCL5 | 0.085 | 0.114 |
| DCN | 0.090 | 0.039 |
| CSTB | 0.093 | 0.136 |
| CDH13 | 0.124 | 0.071 |
